# Complementary Keratoconus Indices Based on Topographical Interpretation of Biomechanical Waveform Parameters: A Supplement to Established Keratoconus Indices

**DOI:** 10.1155/2017/5293573

**Published:** 2017-02-07

**Authors:** Susanne Goebels, Timo Eppig, Stefan Wagenpfeil, Alan Cayless, Berthold Seitz, Achim Langenbucher

**Affiliations:** ^1^Department of Ophthalmology, Saarland University Medical Centre, Kirrberger Strasse 100, Bldg. 22, Homburg, 66421 Saar, Germany; ^2^Department of Experimental Ophthalmology, Saarland University, Kirrberger Strasse 100, Bldg. 22, Homburg, 66421 Saar, Germany; ^3^Institute for Medical Biometry, Epidemiology and Medical Informatics, Saarland University, Campus Homburg, Bldg. 86, Homburg, 66421 Saar, Germany; ^4^Department of Physical Sciences, Open University, Milton Keynes MK7 6AA, UK

## Abstract

*Purpose*. To build new models with the Ocular Response Analyzer (ORA) waveform parameters to create new indices analogous to established topographic keratoconus indices.* Method*. Biomechanical, tomographic, and topographic measurements of 505 eyes from the Homburger Keratoconus Centre were included. Thirty-seven waveform parameters (WF) were derived from the biomechanical measurement with the ORA. Area under curve (ROC, receiver operating characteristic) was used to quantify the screening performance. A logistic regression analysis was used to create two new keratoconus prediction models based on these waveform parameters to resample the clinically established keratoconus indices from Pentacam and TMS-5.* Results*. ROC curves show the best results for the waveform parameters p1area, p2area, *h*1, *h*2, dive1, mslew1, aspect1, aplhf, and dslope1. The new keratoconus prediction model to resample the Pentacam topographic keratoconus index (TKC) was WF_TKC_ = −4.068 + 0.002 × p2area − 0.005 × dive1 − 0.01 ×* h*1 − 2.501 × aplhf, which achieves a sensitivity of 90.3% and specificity of 89.4%; to resample the TMS-5 keratoconus classification index (KCI) it was WF_KCI_ = −3.606 + 0.002 × p2area, which achieves a sensitivity of 75.4% and a specificity of 81.8%.* Conclusion*. In addition to the biomechanically provided Keratoconus Index two new indices which were based on the topographic gold standards (either Pentacam or TMS-5) were created. Of course, these do not replace the original topographic measurement.

## 1. Introduction

Keratoconus is an ectatic corneal disorder, in which the cornea assumes a conical shape due to deficits of structural integrity. This loss of structural components results in a thinning and protrusion of the cornea [1], inducing irregular astigmatism and myopia and leading to a mild to marked deterioration of visual acuity [2]. Vision-specific quality of life is significantly affected by keratoconus [3]. In most cases the disease is bilateral and mostly diagnosed in the second or third decade of life with an incidence of 55 : 100,000 [2, 4, 5].

In current clinical practice, corneal tomography or topography systems are being used to assist the examiner in classifying and monitoring the disease. Since 2005 the evaluation of biomechanical properties has extended the diagnostic spectrum [6].

The Ocular Response Analyzer (ORA, Reichert, Depew, USA) provides data about corneal hysteresis, corneal resistance factor, and intraocular pressure and—in an additional keratoconus tool—a Keratoconus Match Index and Keratoconus Match Probabilities Index.

The ORA software provides a “cornea corrected intraocular pressure” (IOPcc), which is based on the pneumotonometer measurement of the ORA and calibrated to the classical intraocular pressure measurement which is derived with a Goldmann applanation tonometer (IOPg), which is currently the gold standard in a clinical setup.

As topography is the gold standard in keratoconus screening, data from the TMS-series or the Pentacam have primarily been used for helping to diagnose keratoconus.

Our aim was to create a “topography correlated Keratoconus Index” analogous to the IOPg, which is based firstly on TMS data and secondly on Pentacam data.

## 2. Patients and Methods

This retrospective study was conducted at the Department of Ophthalmology, Saarland University Medical Centre in Homburg/Saar (UKS), Germany. The study adhered to the tenets of the Declaration of Helsinki. Each subject provided informed written consent to participate in this research study.

Patients were recruited from the database of the Homburg Keratoconus Centre (HKC). In the HKC, patients with keratoconus were included. The HKC includes also patients without obvious corneal abnormalities but with dysfunction of the thyroid gland, as thyroid disorders are suspected of leading to corneal biomechanical changes [7]. The diagnostic methods and goals of the HKC have been previously described in detail [8–10].

Inclusion criteria for the calculations for this study were no ocular surgery, no other ocular diseases than keratoconus, complete data of the complete ophthalmological examination, including tomography measurements using Scheimpflug imaging (Pentacam, Oculus Optikgeräte GmbH, Wetzlar, Germany), Placido disk video topography (TMS-5, Tomey Corporation, Nagoya, Japan), and biomechanical examination using the Ocular Response Analyzer (ORA).

The classification into normal or keratoconus patients was based on the Keratoconus Index (KCI ≥ 5%) by TMS-5 and on the Topographic Keratoconus Classification (TKC ≥ 1) by Pentacam. An additional control group of normal eyes was not established, as the main aim is to compare screening results of different devices and not to test the screening capacity of the devices.

The HKC includes in total 8195 data sets, of which each eye and each measurement made at a different time point is a separate data set. Selecting only the most current measurement of only one eye of a patient and with complete examination we were able to evaluate the data of 505 patients.

### 2.1. Ocular Response Analyzer

**Figure 1:**
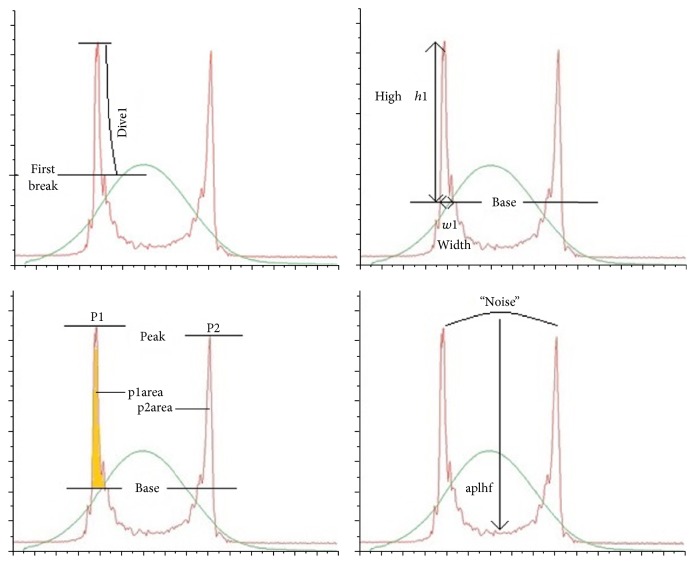
Explanation of the parameters which were used for the model for WF_TKC_ and WF_KCI_: dive1 as maximum single decrease in the fall of peak 1 or longest continuous line without a break, height 1 (*h*1) as the distance from the lowest to the highest point in peak 1, p2area as area under the second peak, and aplhf as high frequency “noise” in region between peaks.

The ORA acts as an air-puff tonometer and assesses the kinetics of the cornea during inward and outward movement. The deformation dynamics of the cornea, such as height, slope, and width, were described with more than 40 parameters, so-called waveform parameters. The Keratoconus Match Index (KMI) is a composite value of the seven waveform parameters (CRF, p2area, dslope1,* h*2, p2area1,* h*21, and waveform score). The explanations of the waveform parameters are presented in Figure 1 and the Supplementary Table, available online at https://doi.org/10.1155/2017/5293573.

### 2.2. Statistical Analysis

Statistical analysis was performed using SPSS software (SPSS version 19.0, International Business Machines Corporation, New York, United States of America). The binary decision for healthy versus keratoconus was tested.

The following steps were performed using the TMS-5 based and using the Pentacam based keratoconus index as reference to create a waveform index based on KCI (WF_KCI_) and a second index based on TKC (WF_TKC_).

1. *Finding the relevant waveform parameters*. Receiver operating characteristic (ROC) curves were calculated for all 37 waveform parameters and quantified using the area under the curve (AUC) for each parameter. The 8 values with the AUC closest to 1 or 0 were selected for the subsequent analysis. A descriptive evaluation of data was performed using maximum, minimum, median, and mean along with respective standard deviation of the keratoconus and the healthy group.
2. *Proving the predictability*. Discriminant function analysis was used to prove the predictability of the selected values. Values which were considered as not significant were excluded from further calculations.
3. *Creating a model*. Logistic regression analysis was used to investigate the discriminatory effect of prespecified model parameters on the outcome for normal (0) versus keratoconus (1) eyes. Model covariate effects were estimated with maximum likelihood and respective *p* values were given. The resulting model predicting probability of keratoconus was used to discriminate between the groups.
4. *Proof of concept.* To validate the prediction formulae derived from the logistic regression analysis, the AUC was calculated. The optimum cutoff points were calculated using Youden's* J* statistic as the sum of sensitivity and specificity minus 1 [11]. *J* lies within a range from −1 to +1. A diagnostic test can be considered to yield reasonable results for positive values of* J*. Higher values indicate better performance of the diagnostic test [11].
5. In absence of an external control group the validation of the logistic regression was done using cross validation. The study population was split randomly into 2 groups; for both groups a prediction model was derived and validation was done with both models for each study subpopulation.

## 3. Results

Five hundred and five eyes were selected for this study. The eyes were classified into keratoconus and normal using TKC and KCI. With TKC/KCI, 73.3%/67.3% was classified as keratoconus, respectively.  Table 1 summarizes the ROC curve analysis results for all new waveform parameters.

## 4. Results Based on the Keratoconus Index TKC

For further calculations we selected the values with the highest AUC: p1area, p2area,* h*1,* h*2, dive1, mslew1, aspect1, and aplhf. All selected parameters correlated with each other.

Table 2(a) shows descriptive statistics such as minimum, maximum, median, and mean with standard deviation of the selected parameters.

With logistic regression analysis, only four of these values were found to be statistically significant (*p* < 0.05): p2area, dive1,* h*1, and aplhf.

Logistic regression (Table 3(a)) resulted in a model based on TKC of

(1) WFTKC=−4.068+0.002×p2area−0.005×dive1−0.01×h1−2.501×aplhf.

For WF_TKC_ we found an AUC with 0.944. The *J*-Index was highest with 0.78 at the threshold of 0.32. With this threshold, sensitivity was 90.3% and specificity was 89.4%.

## 5. Results Based on the Keratoconus Index KCI

For further calculations, we selected the values with the highest AUC:

p1area, p2area,* h*1,* h*2, dive1, mslew1, aspect1, aplhf, and dslope1.

All selected parameters correlated with each other.

Table 2(b) shows descriptive statistics such as minimum, maximum, median, and mean with standard deviation of the selected parameters.

With logistic regression analysis, only p2area was statistically significant (*p* < 0.05).

Logistic regression (Table 3(b)) resulted in a model based on KCI of

(2) $$\begin{array}{c} {WF}_{KCI}=-3.606+0.002\times p2area. \end{array}$$

With the model for WF_KCI_ AUC is 0.821. The* J*-Index was highest with 0.57 at the new threshold of 0.36. With this threshold, sensitivity is 75.4% and specificity is 81.8%.

**Figure 2:**
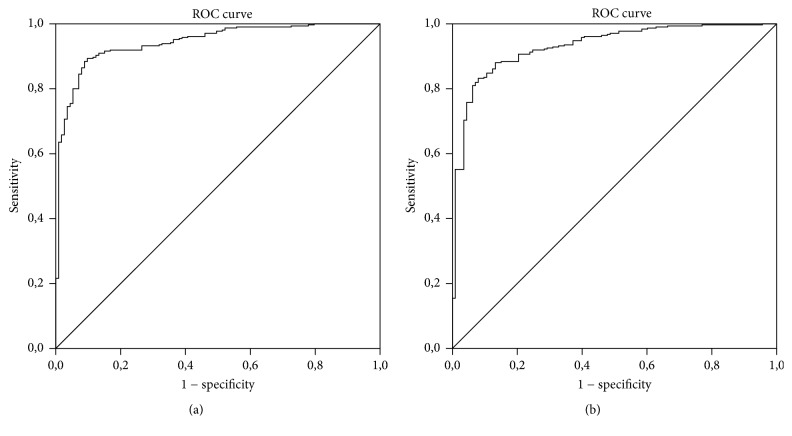
Receiver operating characteristic curves for the new models based on TKC: WF_TKC_ (a) and based on KCI: WF_KCI_ (b). With the model for WF_TKC_ the area under the curve (AUC) is 0.944. With the model for WF_KCI_ AUC the area is 0.821.

Figures 2(a) and 2(b) show both ROC curves for WF_TKC_ and WF_KCI_.

**Figure 3:**
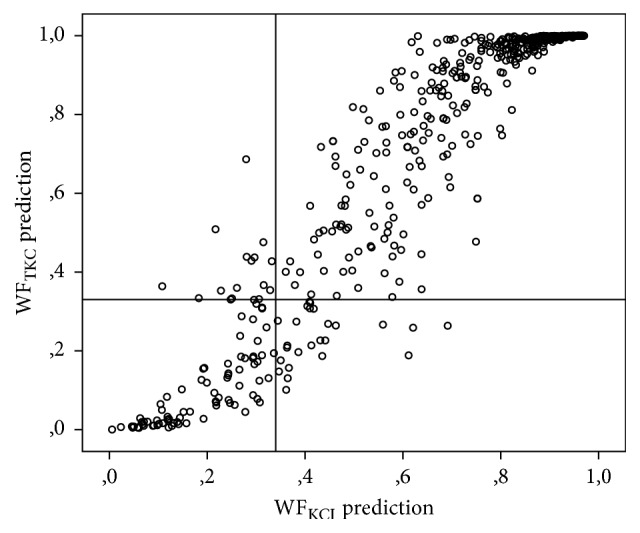
Congruence of WF_TKC_ and WF_KCI_. The upper right and lower left side show identical decisions; the upper left and lower right side show the different decisions of WF_TKC_ and WF_KCI_.

In Figure 3 the congruence of WF_TKC_ and WF_KCI_ is demonstrated. In the upper right and lower left side of the figure identical classifications of the new models are shown, and on the upper left and lower right side the disagreement between WF_TKC_ and WF_KCI_ is demonstrated.

## 6. Cross Validation to Prove the Newly Derived Models

The data set was split into group A with *n* = 248 patients and into group B with *n* = 257 patients.

According to TKC 73.7%/72.8% of these patients were classified as keratoconus (group A/B, respectively). Using group A as reference group, the waveform parameters p2area, dive1,* h*1, and aplhf achieved significant values of <0.001. The model found based on group A was WV_TKC  (Group  A)_ = −3.954 + 0.002 *∗* p2area − 0.004 *∗* dive1 + 0.01 *∗ h*1 − 2.382 *∗* aplhf. For WF_TKC  (Group  A)_ we found an AUC with 0.93. With a threshold of 0.5, the sensitivity was 91.6% and the specificity was 65.5%.

Using group B as reference group the waveform parameters p2area, dive1,* h*1, and aplhf achieved significant values of <0.001. The model found based on group A was WV_TKC  (Group  B)_ = −4.082 + 0.002 *∗* p2area − 0.006 *∗* dive1 + 0.01 *∗ h*1 − 2.593 *∗* aplhf. For WF_TKC  (Group  B)_ we found an AUC with 0.958. With a threshold of 0.5, the sensitivity was 93.6% and the specificity was 82.8%.

According to KCI 69.2%/65.19% of these patients were classified as keratoconus (group A/B, respectively).

Using group A as reference group to find WF_KCI_ the significance of the waveform parameter p2area was <0.001. The model found based on group A was WV_KCI  (Group  A)_ = −3.497 + 0.002 *∗* p2area. For WF_KCI  (Group  A)_ we found an AUC with 0.85. With a threshold of 0.5, the sensitivity was 92.3% and the specificity was 50.8%.

Using group B as reference group to find WF_KCI_ the significance of the waveform parameter p2area was <0.001. The model found based on group A was WV_KCI  (Group  A)_ = −3.506 + 0.002 *∗* p2area. For WF_KCI  (Group  A)_ we found an AUC with 0.865. With a threshold of 0.5, the sensitivity was 91.0% and the specificity was 60.6%.

## 7. Discussion

The Ocular Response Analyzer was launched onto the market in 2005 as the first device which allows in vivo evaluation of biomechanical behaviour of the eye [6]. The software version 3.0 or higher provides a keratoconus screening tool with keratoconus indices [12]. The Keratoconus Match Index (KMI) represents the similarity of the waveform from an eye to the average waveform characteristics of various Keratoconus eyes. The data for this reference data base were collected in four different clinical centres, where diagnosis of keratoconus was performed clinically and with different topographic or tomographic devices.

In this study we designed complementary keratoconus indices supplementary to the KMP of ORA. As different devices were used for the reference data set to evaluate keratoconus, we derive one index for each reference device. One is based on the Scheimpflug assisted keratoconus screening (WF_TKC_) and the other is based on the topographic assisted keratoconus screening (WF_KCI_). This is in analogy to the “cornea corrected pressure” (IOPcc) or the “Goldmann correlated pressure” (IOPg), which is provided in the ORA software.

The area under peak 2 showed the best AUC and yielded the best separation between normal and keratoconus patients in both models. Finally, this is the only relevant waveform parameter for the KCI based new index. For the TKC based index the* h*1, aplhf, and dive1 were also included in the model in order to discriminate normal from keratoconus eyes. This is in accordance with the typical findings in waveform changes in keratoconus eyes: the amplitude of peaks 1 and 2 decreases, the peaks become thin and sharp, and additional spikes are found in the peaks, as well as “noise” in between the peaks (User's Guide Ocular Response Analyzer). Although we found a high AUC for both models, the sensitivity and specificity of WF_KCI_ were low. This suggests that biomechanical properties better reflect Pentacam than TMS-5 based keratoconus screening. This supports our previous findings [9, 10].

A few previous studies have dealt with the additional waveform parameters based on a small number of patients. Luz et al. investigated 112 normal eyes and 41 keratoconic eyes. Keratoconus diagnosis was based on clinical data and Pentacam Scheimpflug measurements. They found statistically significant differences between keratoconic and normal eyes in all 41 waveform parameters except IOPcc and W1. The area under the ROC curve was larger than 0.85 for 11 parameters, especially p2area and p1area (also p2area1,* h*1,* h*11, p1area1, CRF, dive2,* h*2,* h*21, and CH). But significant overlap was noted and even for the new parameters in the present study no cutoff value could be established with a sufficient sensitivity and specificity [13]. Mikielewicz et al. tested the use of the parameter in 48 healthy and 54 keratoconic patients. Diagnosis was based on Amsler-Krumeich-criteria using ROC curves. They found out that the best parameters to discriminate between keratoconus and control eyes were CRF, CH, p1area, p2area,* h*1,* h*2, dive2, p1area1, p2area2, and time1 [14].

Only a few studies have dealt with the keratoconus indices which were provided by the ORA [15, 16]. Labiris et al. evaluated the screening capability of the KMI and KMP indices in keratoconus suspect eyes and their association with a series of Scheimpflug camera-derived keratoconus indices. They did not find any correlation between KMI and any Scheimpflug index measured in any group. They found that the KMI differed significantly between control eyes and keratoconus suspect eyes with an overall predictive accuracy of 94%. The results of KMP did not correlate with the study groups. A high percentage of suspect eyes in the normal population as well as a high percentage of KMP-derived normal eyes in the keratoconus suspect group were found. They conclude that this high percentage of suspect eyes in the normal population reflects an insufficiency of the index that limits the discrimination between normal eyes and those with suspected keratoconus [15]. In a second paper Labiris et al. evaluated KMI and KMP in 114 keratoconic eyes, which were diagnosed using Amsler criteria. For KMI they found a high screening capacity in keratoconic eyes (all stages) with overall predictive accuracy of 97.7% [16]. Also here KMP did not correlate with the study groups.

In a previous paper, we evaluated the congruence of keratoconus classification based on clinical, topographic, tomographic, and biomechanical indices. This was the first study which dealt with the different keratoconus indices of different devices. The congruence of keratoconus classification was found to be quite poor with these techniques [9]. But as the different devices are based on different keratoconus attributes, such as biomechanical, tomographic, or topographic measures and clinical aspects, they are not expected to be interchangeable.

In conclusion, currently, the Ocular Response Analyzer should be used as second line, as a complimentary device for detecting keratoconus.* In addition to the proprietary biomechanical Keratoconus Match Index we created two new indices, which are based on* Pentacam (TKC) and TMS-5 (KCI) measurements: WF_TKC_ = −4.068 + 0.002 × p2area − 0.005 × dive1 − 0.01 ×* h*1 − 2.501 × aplhf and WF_KCI_ = −3.606 + 0.002 × p2area, which achieve a high sensitivity and specificity. Of course, these new indices do not replace the original topographic measurement.

## Supplementary Material

Explanation of the 37 additional waveform parameters. The different suffixes 1, 2, 11, 21 refer, respectively, to the upper 75% of the first (1) or second (2) peak, or to the upper 50% of the first (11) or the second (21) peak.

## Figures and Tables

**Table 1:** Area under curve (AUC) for all 37 new waveform parameters. The values with the AUC closest to 1 or 0 were selected for further calculation.

	AUC based on TKC	AUC based on KCI
p1area	0.11	0.20
p2area	0.07	0.18
*h*1	0.11	0.21
*h*2	0.15	0.25
*w*1	0.47	0.46
*w*2	0.33	0.35
aspect1	0.18	0.27
aspect2	0.33	0.40
dslope1	0.20	0.29
dslope2	0.37	0.42
uslope1	0.21	0.31
uslope2	0.31	0.38
path1	0.67	0.68
path2	0.78	0.72
slew1	0.212	0.33
slew2	0.32	0.40
mslew1	0.17	0.29
mslew2	0.28	0.37
aindex	0.25	0.30
bindex	0.4	0.47
dive1	0.16	0.26
dive2	0.21	0.29
aplhf	0.84	0.74
p1area1	0.12	0.2
p2area1	0.08	0.18
*h*11	0.11	0.21
*h*21	0.15	0.25
*w*11	0.41	0.340
*w*21	0.27	0.31
aspect11	0.23	0.32
aspect21	0.37	0.43
dslope11	0.28	0.35
dslope21	0.40	0.44
uslope11	0.22	0.33
uslope21	0.32	0.42
path11	0.64	0.66
path21	0.70	0.67

AUC: area under the curve.

TKC: Topographic Keratoconus Classification derived from Pentacam.

KCI: keratoconus classification index derived from TMS-5.

**(a):** 

Covariate	Mean value		Median		Minimum		Maximum	
	Normal	Kc	Normal	Kc	Normal	Kc	Normal	Kc
p1area	4007 ± 1122	2113 ± 1119	3818	1916	1539	154.16	8388	7001
p2area	2694 ± 714	1289 ± 638	2703	1219	666.3	67.75	5454	3891
Aspect1	19.81 ± 5.29	12.01 ± 6.9	19.75	10.88	4.68	0.57	41.87	36.29
*H*1	433.4 ± 98.10	245.9 ± 119.69	432.94	241.78	121.68	27.93	649.88	635.06
*H*2	350.4 ± 87.90	212.8 ± 98.26	353.81	213.84	78.56	20.25	576.38	563.44
Dive1	379.7 ± 125.01	209 ± 118.34	391.5	202.38	23.25	2.75	615.78	568.0
Mslew1	119.7 ± 33.65	75.30 ± 38.09	112.25	72.38	29.25	6.75	213.0	258.25
aplhf	1.15 ± 0.23	1.68 ± 0.60	1.1	1.5	0.7	0.9	1.8	5.2

**(b):** 

Covariate	Mean value		Median		Minimum		Maximum	
	Normal	Kc	Normal	Kc	Normal	Kc	Normal	Kc
p1area	3514 ± 1262	2137 ± 1131	3585	2011	508	154	8388	7001
p2area	2309 ± 867	1325 ± 684	2372	1222	255	67.75	5454	4074
Aspect1	17.34 ± 6.33	12.15 ± 6.78	18.21	10.94	1.47	0.57	33.78	36.29
*H*1	376 ± 121	250 ± 120	395.16	244	36.82	27.93	650	635
*H*2	304 ± 103	215 ± 98	320.95	214	22.79	20.25	555	56
Dive1	319 ± 139	208 ± 118	345	203	8.25	2.75	615.78	568
Mslew1	105 ± 40.44	76.34 ± 36.57	104	73	8.0	6.75	242.75	206
aplhf	1.310 ± 0.4909	1.68 ± 0.59	1.2	1.5	0.8	0.8	4.4	5.2
dslope1	25.19 ± 9.81	17.77 ± 10.66	26.33	15.48	1.80	0.93	62.66	63.63

**(a):** 

Variable	Estimated effect	*p* value
Constant	−4.01	0.002
p2area	0.02	<0.01
dive1	0.01	0.002
*h*1	−0.005	0.043
aplhf	−2.501	0.001

TKC: Topographic Keratoconus Classification.

**(b):** 

Variable	Estimated effect	*p* value
Constant	−3.606	<0.01
p2area	0.002	<0.01

KCI: keratoconus classification index.
