# Supplementary material for: Complementary Keratoconus Indices Based on Topographical Interpretation of Biomechanical Waveform Parameters: A Supplement to Established Keratoconus Indices
Source: Comput Math Methods Med. 2017 Feb 7;2017:5293573. doi: 10.1155/2017/5293573 (PMC5320073; doi:10.1155/2017/5293573)
Supplement: Supplementary file 1 — Explanation of the 37 additional waveform parameters. The different suffixes 1, 2, 11, 21 refer, respectively, to the upper 75% of the first (1) or second (2) peak, or to the upper 50% of the first (11) or the second (21) peak. [file 5293573.f1.pdf]

Supplementary table: Explanation of the 37 additional waveform parameters**.** *The different suffixes 1, 2, 11, 21 refer, respectively, to the* upper 75% of *the first (1) or second (2) peak, or to the upper 50% of the first (11) or the second (21) peak.*

	**Upper 75% of peak height**	**Upper 50% of peak height**	
Area	p1area<br>p2area	p1area1<br>p2area1	Area under peak 1 or peak 2
Height	h1<br>h2	h11<br>h21	Height from the lowest to the highest point in peak
width	w1<br>w2	w11<br>w21	Width at the "base" of the peak region
Aspect ratio	aspect1<br>aspect2	aspect11<br>aspect21	Quotient of width to height
Slope	dslope1<br>dslope2	dslope11<br>dslope21	rate of decease from base to peak (downward slope)
	uslope1<br>uslope2	uslope11<br>uslope21	rate of increase from base to peak<br>(upward slope)
Slew rate	slew1<br>slew2		Quotient of dive to width
	mslew1<br>mslew2		maximum single increase in the rise of peak 1 and 2<br>(longest continuous line without a break)
Path	path1<br>path2	path11<br>path21	absolute value of path length around peak 1 and 2
Irregularity	aindex<br>bindex		"smoothness" of peak 1, peak 2<br>number of times the peak changes direction

Dive	dive1<br><br>dive2		maximum single increase in the fall of peak 1 and 2<br><br>(longest continuous line without a break)
High frequency	aplhf		high frequency “noise” in region between peaks
